# Molecular basis for phenotypic similarity of genetic disorders

**DOI:** 10.1186/s13073-019-0641-y

**Published:** 2019-04-23

**Authors:** Vijay Kumar Pounraja, Santhosh Girirajan

**Affiliations:** 1 Bioinformatics and Genomics Program, The Huck Institutes of the Life Sciences, Pennsylvania State University, University Park, PA 16802 USA; 2 Department of Biochemistry and Molecular Biology, Pennsylvania State University, University Park, PA 16802 USA; 3 Department of Anthropology, Pennsylvania State University, University Park, PA 16802 USA

## Abstract

The contribution of distinct genes to overlapping phenotypes suggests that such genes share ancestral origins, membership of disease pathways, or molecular functions. A recent study by Liu and colleagues identified mutations in *TCF20,* a paralog of *RAI1*, among individuals manifesting a novel syndrome that has phenotypes similar to those of Smith-Magenis syndrome (a disorder caused by disruption of *RAI1*). This study highlights how structural similarity among genes contributes to shared phenotypes, and shows how this relationship can contribute to our understanding of the genetic basis of complex disorders.

## Paradigms for establishing shared genetic etiology

Establishing the association between genotype and phenotype is the central element for most genetic analyses of complex disorders. Several of these disorders are characterized by genetic heterogeneity, where disruption of a variety of distinct genes can cause similar phenotypes. This heterogeneity can occur as the result of several common evolutionary and functional properties, including shared ancestral origins, protein sequence similarity, overlapping molecular functions, or membership of the same pathways. For example, disruption of *PKD1* and *PKD2*, two polycystin protein genes that share four transmembrane protein domains and interact with each other, can independently lead to polycystic kidney disease [1]. Another classic example is the presence of a common constellation of clinical features in Bardet-Biedl syndrome. These features include rod-cone dystrophy, obesity, hypogonadism, and renal anomalies, among individuals with recessive mutations in any of the cilia-formation genes, such as *BBS1* and *BBS2* [2]. Similarly, *EHMT1* and *MBD5* cause specific intellectual disability-associated disorders and interact within a chromatin-modification network module, highlighting how epigenetic defects may underlie cognitive deficits [3]. Further, mutations in gap junction proteins *GJB2* and *GJB6*, which interact to form heteromeric complexes, both result in deafness [4]. Therefore, investigating functional and molecular similarities among genes that contribute to related phenotypes can provide a broader framework to help establish the etiologies of complex disorders.

## Functional relatedness translating to shared phenotypes

Genomic studies on large cohorts of affected individuals have identified hundreds of genes that may be involved in the etiology of developmental disorders. Several of these studies have unraveled specific genes that are associated with rare Mendelian disorders, whereas others have identified numerous genes that contribute to similar disorders with shared phenotypes. In a recent study, Liu and colleagues analyzed exome sequencing and chromosomal microarray data and identified pathogenic mutations in *TCF20* in 32 affected individuals from 31 unrelated families [5]. *TCF20* encodes an SPRE-binding transcription factor that is strongly expressed in pre-migratory neural crest cells and is known to influence other transcription factors [6]. Although *TCF20* has been previously associated with autism, intellectual disability, and related phenotypes, the authors of this study performed a deeper assessment of phenotypes and identified a pattern of features that were reminiscent of Smith-Magenis syndrome (SMS), a rare disorder caused by disruption of *RAI1* (encoding the retinoic acid induced 1 protein). Like children with SMS, patients with *TCF20* mutations presented a core set of features including facial dysmorphology, hypotonia, seizures, and sleep disturbance.

Liu and colleagues found that commonalities in gene structure and function between *TCF20* and *RAI1* could explain the shared core clinical features and molecular effects [5]. In fact, *TCF20* shares several essential protein domains with *RAI1*, including N-terminal transactivation domains, zinc-finger-like plant homeodomains (PHD), and nuclear localization signal domains [6]. The high sequence homology and conservation of specific domain combinations between *TCF20* and *RAI1* is attributed to a gene duplication event that occurred during early vertebrate evolution [5]. For example, the chromatin-binding PHD domain is highly conserved in both *TCF20* and *RAI1*, and a patient with a missense mutation in the PHD domain of *TCF20* presented with strong SMS-like features [5]. In fact, several PHD domain-containing genes are involved in chromatin modification and transcriptional regulation functions, and are therefore relevant not only to SMS and *TCF20*-associated disorders but also to several other disorders, including *NSD1* and Sotos syndrome, *CREBBP* and Rubinstein-Taybi syndrome, *DPF2* and Coffin-Siris syndrome, and *KMT2D* and Kabuki syndrome [7].

This study adds *TCF20* to a growing list of genes that cause SMS-like phenotypes and to the list of disorders that should be considered in differential diagnosis. In fact, previous studies on individuals who presented with features typical of SMS but did not carry *RAI1* mutations found that these individuals had mutations in: *MBD5*, which were associated with a set of SMS-like neurodevelopmental features and with autism; *EHMT1,* the causative gene for Kleefstra syndrome; *PHF21A,* which is associated with Potocki-Shaffer syndrome; or *TCF4*, which is associated with Pitt-Hopkins syndrome. Furthermore, Loviglio and colleagues [8] identified *POGZ*, *BRD2*, *KDM5C*, and *ZBTB17* within an *RAI1*-associated network, and Berger and colleagues [9] identified *DEAF1* and *IQSEC2*, whose disruption resulted in phenotypes overlapping with those associated with SMS. As the network of genes associated with SMS-like phenotypes grows, it is likely that some of these genes traverse pathways related to common disorders including autism, cognitive defects, and sleep abnormalities.

## A protein domain-centric view of disease

**Fig. 1:**
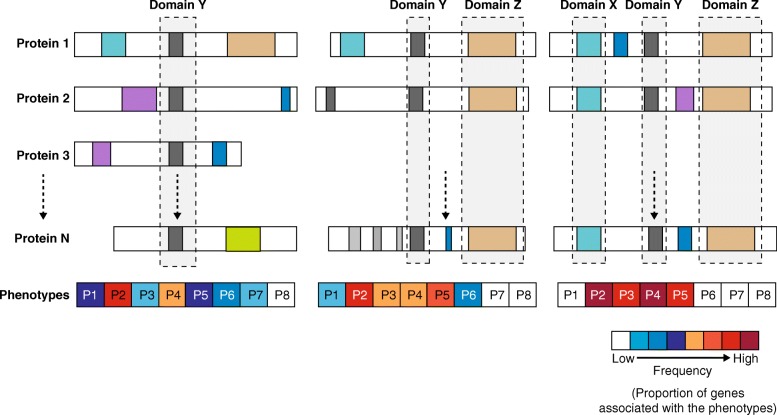
A domain-centric view of disease. The figure shows a model for how genes that share a combination of domains are more likely to show a similar set of phenotypes. In this model, genes that code for proteins 1 to N share various protein domains, including domains X, Y, and Z, and their disruption leads to phenotypes P1–P8. Frequency is defined as the number of genes that are associated with a phenotype out of all the genes that share the domain or combination of domains. Specificity for the manifestation of certain phenotypes increases as the number of shared domains increases. In this case P2–P5 show increased frequency as the number of shared domains increases while the other phenotypes are no longer associated with the increasingly complex domain combination

With the emergence of the deep phenotyping and genome sequencing of large cohorts of individuals as part of clinical care and precision-medicine initiatives, future studies will expand upon the approach outlined by Liu and colleagues to identify novel instances of phenotypic convergence among individuals carrying mutations in functionally related genes [5]. One approach would be to interrogate whether genes that share common protein domains confer risks for similar phenotypes (Fig. 1). For example, the PDZ-domain-containing *SHANK* and *NLGN* family of genes are involved in synaptic signaling and are associated with autism [10]. Nevertheless, the presence of a single domain in a gene may not always be predictive of a specific phenotype, because the ultimate biological effects of that gene could also depend on the presence of other functional domains. As observed for *RAI1* and *TCF20*, genes that share combinations of domains could confer greater specificity for a particular set of phenotypes. This could potentially explain why other genes that both encode proteins that contain PHD domains, such as *NSD1* and *KMT2B*, and contribute to neurodevelopmental disorders do not share a full set of phenotypic associations with SMS [7]. Further studies could also search for an over- or under-representation of genes with a specific composition of conserved protein domains in one or more phenotypic categories. Liu and colleagues carve an exciting paradigm for identifying the functional relatedness of genes on the basis of shared phenotypes, which could potentially help to refine common networks and pathways for neurodevelopmental disorders and other complex genetic diseases [5].

## Conclusions

Studies on larger populations of affected individuals will continue to identify associations between diseases and genes that are associated with distinct categories of biological pathways, genetic networks, and molecular mechanisms. The discovery of disease-associated genes on the basis of shared domains and evolutionary history, as described by Liu and colleagues [5], could be used to further refine connections between genes that contribute to related disorders and to provide mechanistic specificities for genes within these broader functional categories.

## Abbreviations

PHD: Plant homeodomain
SMS: Smith-Magenis syndrome
